# Preparatory Body State before Reacting to an Opponent: Short-Term Joint Torque Fluctuation in Real-Time Competitive Sports

**DOI:** 10.1371/journal.pone.0128571

**Published:** 2015-05-29

**Authors:** Keisuke Fujii, Daichi Yamashita, Tetsuya Kimura, Tadao Isaka, Motoki Kouzaki

**Affiliations:** 1 Research Center of Health Physical Fitness and Sports, Nagoya University, Nagoya, Japan; 2 Research Fellow of the Japan Society for the Promotion of Science, Tokyo, Japan; 3 Japan Institute of Sports Sciences, Tokyo, Japan; 4 Graduate School of Human Development and Environment, Kobe University, Hyogo, Japan; 5 Faculty of Sports and Health Science, Ritsumeikan University, Shiga, Japan; 6 Graduate School of Human and Environmental Studies, Kyoto University, Kyoto, Japan; VU University Amsterdam, NETHERLANDS

## Abstract

In a competitive sport, the outcome of a game is determined by an athlete’s relationship with an unpredictable and uncontrolled opponent. We have previously analyzed the preparatory state of ground reaction forces (GRFs) dividing non-weighted and weighted states (i.e., vertical GRFs below and above 120% of body weight, respectively) in a competitive ballgame task and demonstrated that the non-weighted state prevented delay of the defensive step and promoted successful guarding. However, the associated kinetics of lower extremity joints during a competitive sports task remains unknown. The present study aims to investigate the kinetic characteristics of a real-time competitive sport before movement initiation. As a first kinetic study on a competitive sport, we initially compared the successful defensive kinetics with a relatively stable preparatory state and the choice-reaction sidestep as a control movement. Then, we investigated the kinetic cause of the outcome in a 1-on-1 dribble in terms of the preparatory states according to our previous study. The results demonstrated that in successful defensive motions in the non-weighted state guarding trial, the times required for the generation of hip abduction and three extension torques for the hip, knee, and ankle joints were significantly shortened compared with the choice-reaction sidestep, and hip abduction and hip extension torques were produced almost simultaneously. The sport-specific movement kinetics emerges only in a more-realistic interactive experimental setting. A comparison of the outcomes in the 1-on-1 dribble and preparatory GRF states showed that, in the non-weighted state, the defenders guarded successfully in 68.0% of the trials, and the defender’s initiation time was earlier than that in the weighted state (39.1%). In terms of kinetics, the root mean squares of the derivative of hip abduction and three extension torques in the non-weighted state were smaller than those in the weighted state, irrespective of the outcome. These results indicate that the preparatory body state as explained by short-term joint torque fluctuations before the defensive step would help explain the performance in competitive sports, and will give insights into understanding human adaptive behavior in unpredicted and uncontrolled environments.

## Introduction

Humans interact by changing their actions, perceiving other’s actions, and then quickly searching and executing optimized solutions to various problems [1, 2] in an unpredictable environment [3]. These adaptive, intelligent, cognitive, and motor-control processes are created by our multi-joint body movements [4, 5]; however, these adaptive movements caused by the human motor system are still unclear because infinite solutions exist (e.g., joint positions and angles) to perform a certain movement [3]. Understanding adaptive human movements in an unpredictable environment will give greater insights into the science of human behavior [6–8], help prevent or aid in the recovery of motor function impairment [9, 10], and facilitate multi-joint robotics [11, 12]. Typical examples for adaptive human movements in an unpredictable environment can be observed in ball sports such as basketball [13–16] and tennis [17, 18], and martial arts such as boxing [19, 20] and Japanese kendo [6, 21], where the outcome of the game is determined by an athlete’s relationship with an unpredictable and uncontrolled opponent.

Skilled ballgame players perceive relevant cues to an opponent’s motion [22, 23], decide on the suitable action [24], and begin to move in the appropriate direction [25, 26]. To contribute to our understanding of the process of competitive interaction, researchers captured the motions of both players in the 1-on-1 subphase [13–16]. In this situation, competition dynamics could be considered as a complicated problem for researchers [27, 28] because the two players quickly and predictively interact. To overcome this difficulty, previous studies have described competitive dynamics using a dynamical system approach [17, 29], in which the dynamics are analyzed as a system of pairs of players. However, the influence of an individual player’s cognitive-motor performance on the competitive outcome is still unknown.

In net ball sports [17, 18], martial arts [6, 19], and tag play [30], players quickly change or play in parallel their roles as attackers and defenders. However, in invasive sports such as basketball, an attacker is clearly defined as a player who holds the ball. Our previous study focused on the dribbler’s and defender’s initiation time in 1-on-1 dribble subphase of basketball [13], suggested that the defender mostly moves after the dribbler initiates in order to stop the dribbler. Furthermore, we have focused on the defender’s delay and analyzed the discrete defender’s preparatory state of ground reaction forces (GRFs) for the defender’s step initiation, which divided the non-weighted and weighted states (i.e., peak vertical GRFs below and above 120% of the body weight (BW) before the initiation, respectively) as critical variable to explain the outcome and the defender’s performance in the same sport task [16]. Counterintuitively, the results of the previous study demonstrated that the non-weighted state prevented delay of the defensive step and promoted successful guarding. The effect of preparatory GRF state have been also confirmed by the experimentally controlled choice-reaction sidestepping study in which the line of motion is primarily along the sideways axis, demonstrating that step initiation was delayed in the weighted state [31]. However, the associated mechanical cause of the delay and the preparatory GRF state remains unknown.

The GRF for a player’s maneuver is derived from human multi-joint torques [32, 33]. Researchers have investigated voluntarily-started sidejumps [34] and voluntary [35] and choice-reaction [36] cutting maneuvers. In the study of skills where the environment constantly changes and movements have to be continually adapted [37], the experimental task was simulated using a choice-reaction task [25, 31]. However, movement kinetics such as joint torques in an unpredictable and uncontrolled situation including various opponent maneuvers such as deceptive movements remain unknown. The characteristics of movement kinetics in a competitive sport are sport-specific, i.e., task-constrained according to the rules of the sport, and are assumed to have a short-term fluctuating component from previous actions during an analyzed phase (e.g., outcome-determination phase in 1-on-1 dribble [16]). In the current study, we first compared the choice-reaction sidestep movement [25, 31] and successful defensive motion with a relatively stable preparatory state, i.e., non-large GRF (termed as “non-weighted state”), to investigate the characteristics of sport-specific movement kinetics and the aforementioned short-term fluctuation component as the first kinetic analysis of real-time 1-on-1 competition in sports. This analysis will provide greater insights into movement kinetics involving sudden direction changes combined with the acceleration or deceleration of the body, where non-contact injury in sports (such as anterior cruciate ligament injury) sometimes occurs [35 38].

Additionally, the kinetic cause of performance and outcome in a competitive sport should be revealed because it informs us how an advantage for researchers, coaches and players may be generated from the kinetics perspective in a given sport. In the optimal movement kinetics, humans are assumed to plan to move by minimizing any change in joint torques [39, 40]. Conversely, a greater change in the torque is assumed to result in the lack of efficiency of the intended movement. In addition, in competitive sport maneuvers, the transfer of the center of mass (CoM) is derived from the GRF which is created by human multi-joint torques. Given the above insights, the relationship among performance, preparatory GRF state, and short-term multi-joint torque fluctuation should be investigated. In the present study, we categorized the trial into the outcome (penetrating and guarding) and the defender’s preparatory GRF state (non-weighted and weighted states) in 1-on-1 dribble, and compared the short-term fluctuation of joint torque changes (termed as “preparatory body state”) for the defender’s lower body among the categories. We assumed that the suppression of the fluctuation in the joint torques can be observed in the guarding trial and non-weighted state.

The present study aims to investigate the kinetic characteristics of a real-time competitive sport before the movement initiation. In the current study, we first compared the choice-reaction sidestep movement [25, 31] and successful defensive motions with relatively stable non-weighted state, and hypothesized that sport-specific movement kinetics and a short-term fluctuation component could be observed in the successful defensive motions. Second, we investigated the kinetic cause of performance and outcome, and hypothesized that the suppression of fluctuation in the joint torques could be observed in the guarding trial and the non-weighted state. An evaluation of the short-term fluctuation of joint torques before initiation will help people more substantially understand the kinetics of preparatory states for competitive sports movements where a defender reacts to an attacker.

## Methods

### Participants

In this study, 10 skilled males of a university basketball team (age = 19.2 ± 0.4 years, experience = 8.0 ± 1.8 years [mean ± SD]) participated. The participants provided their written informed consent to participate in this study. The experimental procedures were conducted in accordance with the Declaration of Helsinki and approved by the Local Ethics Committee in the Graduate School of Human and Environmental Studies, Kyoto University (approval number 26-H-12).

### Protocol

Prior to the 1-on-1 dribble task, all participants performed a total of 10 trials of a two-alternative forced choice-reaction sidestep task. This task was chosen because there has been no comparable movement in the previous study with the sport-specific movement. Although players in a contact ball sport also move forward and backward, we selected the common step task in only the lateral direction to allow for comparisons with the results of previous studies [25, 31]. Participants were instructed to take two steps laterally toward one of two light-emitting diodes (LEDs) when they blinked and reach the target with their leading foot at a distance equal to their body height from the initial midline. Reaching performance was calculated on the basis of the lateral trunk displacement described below, according to the rules of basketball, which state that the defenders are allowed to stop the attacker by contact with their trunk [25, 31]. The participants did not perform any preparatory motions before the direction was indicated. Two sets of LEDs were placed 2 m from the participants and set at eye level. First, participants were given a preparatory signal consisting of each set of LED lights blinking three times. Then, either the left or the right set of LEDs was illuminated as a direction signal. After a minimum of five familiarization trials, the participants completed 10 reaction tasks. The orders of the conditions and the stimulus were counterbalanced among all participants. We analyzed 46 successful trials in total.

**Fig 1:**
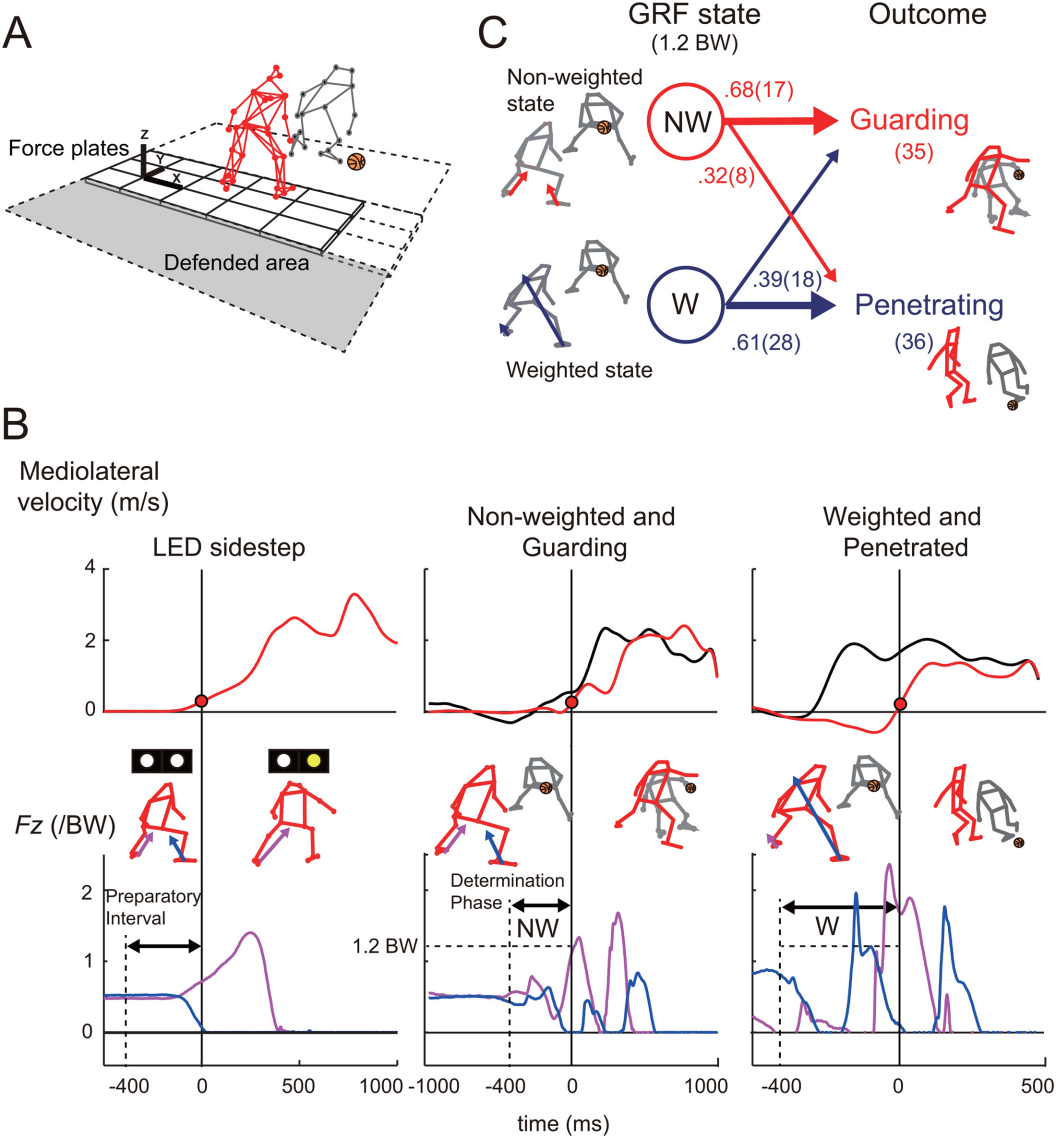
Scene selection. A) Experimental setup and schematic diagram of a basketball defender and dribbler. The objective of the dribbler was to get past the defender and invade the defended area behind the defender. According to the basketball rules, the dribbler was not permitted to cross the sideline and the defender was allowed to stop the dribbler from a head-on position only. (B) Time series of defender’s (red) and dribbler’s (black) mediolateral velocity and vertical ground reaction forces (Fz) of defender’s leading foot (blue) and trailing foot (pink) in a choice-reaction sidestep, a non-weighted (NW) and guarding, and weighted (W) and penetrating trial. The vertical solid and dashed lines are defender’s (0 ms) and choice-reaction signal illumination or dribbler’s initiation times, respectively. The horizontal dashed line for Fz is the criterion for determining non-weighted trials (120% body weight). Preparatory interval is defined as the 400 ms interval before the defender initiated his movement. (C) State transition diagrams with the probabilities of the preparatory GRF state on the outcome of 1-on-1 dribble. We confirmed the outcome probabilities of the non-weighted (NW) and weighted (W) trials. The thickness of arrows represents higher probabilities.

In 1-on-1 dribble task, a dribbler with a basketball and a defender were instructed to play a real-time, 1-on-1 game within a 2.4 × 3.6 m area (mediolateral × anteroposterior space, Fig 1). Players in each pair alternatively assumed the role of a dribbler and a defender (total 16 pairs and 96 trials). There was no basketball goal or scoring opportunity [13, 16]. The experimental task began with a pass from the defender to the dribbler. The objective of the dribbler was to get past the defender and to invade the defended area behind the defender according to standard basketball rules [13]. The defender aimed to stop the dribbler according to the same rules, which allow the defender to stop the dribbler with his/her trunk from a head-on position only. No additional instruction (such as time limit) was given to the dribblers and defenders. All of the trials finished within 10 s.

### Data collection

Kinematic data was acquired using a 3D optical motion capture system with 16 cameras operating at 200 Hz (Raptor-EDigital Real Time System, Motion Analysis Corporation, Santa Rosa, CA, USA), which captured three-dimensional coordinates of participant landmarks. Reflective markers were placed on the landmarks of the participant’s body (right and left side of their heads, shoulders, elbows, wrists, anterior superior iliac spine, hips, heels, toes, medial and lateral knees, and ankles) to obtain CoM, trunk, and lower body segment models (thigh, shank, and foot) using inertial properties of the body segments in Japanese athletes [41]. All raw coordinate data points were smoothed using a fourth-order Butterworth low-pass digital filter (8–12 Hz) using residual analysis [42]. To measure the defenders’ GRFs, 15 force platforms (all 60 × 40 cm) were used with sampling frequency of 1000 Hz (Fig 1A, TF-4060-B, Tec Gihan, Japan). We detected left or right foot contact separately using both our customized program which utilized the location of the force plates and foot markers (ankles, heels, and toes) and visual detection. Both feet were never in contact with the same force platform because of the participants’ wide-base defensive stance. The body segment parameters were interpolated using a spline function from 200 to 1000 Hz for the analyses. This allowed the high spatial resolution necessary to evaluate peak GRFs based on the body movement event (i.e., initiation time) described below.

### Selection and categorization into penetrating and guarding trials

Defending against a dribbler means that the defender can deprive the dribbler of his/her free movement. The 1-on-1 subphase ended only after the dribbler invaded the defended area (defined as penetrating trials), crossed the sideline, stopped dribbling (held the ball) or was deprived of the ball by the defender for any reason, e.g., the dribbler’s poor ball handling. Specifically, guarding against a dribbler indicates that the CoM positional difference between the dribbler and defender and their movement velocity in the mediolateral direction approached zero. One of the authors, a certified basketball coach in Japan (experience: 5 years as coach and 18 years as player), visually judged the penetrating and guarding trials on the basis of the above criteria. The computational criterion was not used because the reflective markers were occasionally invisible toward the end of the trials as the participants moved out of the range of the cameras. This was particularly common in the penetrating trials in the anteroposterior direction and in the guarding trials due to the participants’ contact. The total number of 1-on-1 games (trials) in the present study was 96: 35 guarding trials and 36 penetrating trials (Fig 1C). The remaining 25 trials were excluded due to failures in the recordings of the kinematics or GRFs.

### Analysis

#### Discrete preparatory GRF states and performance variables

**Fig 2:**
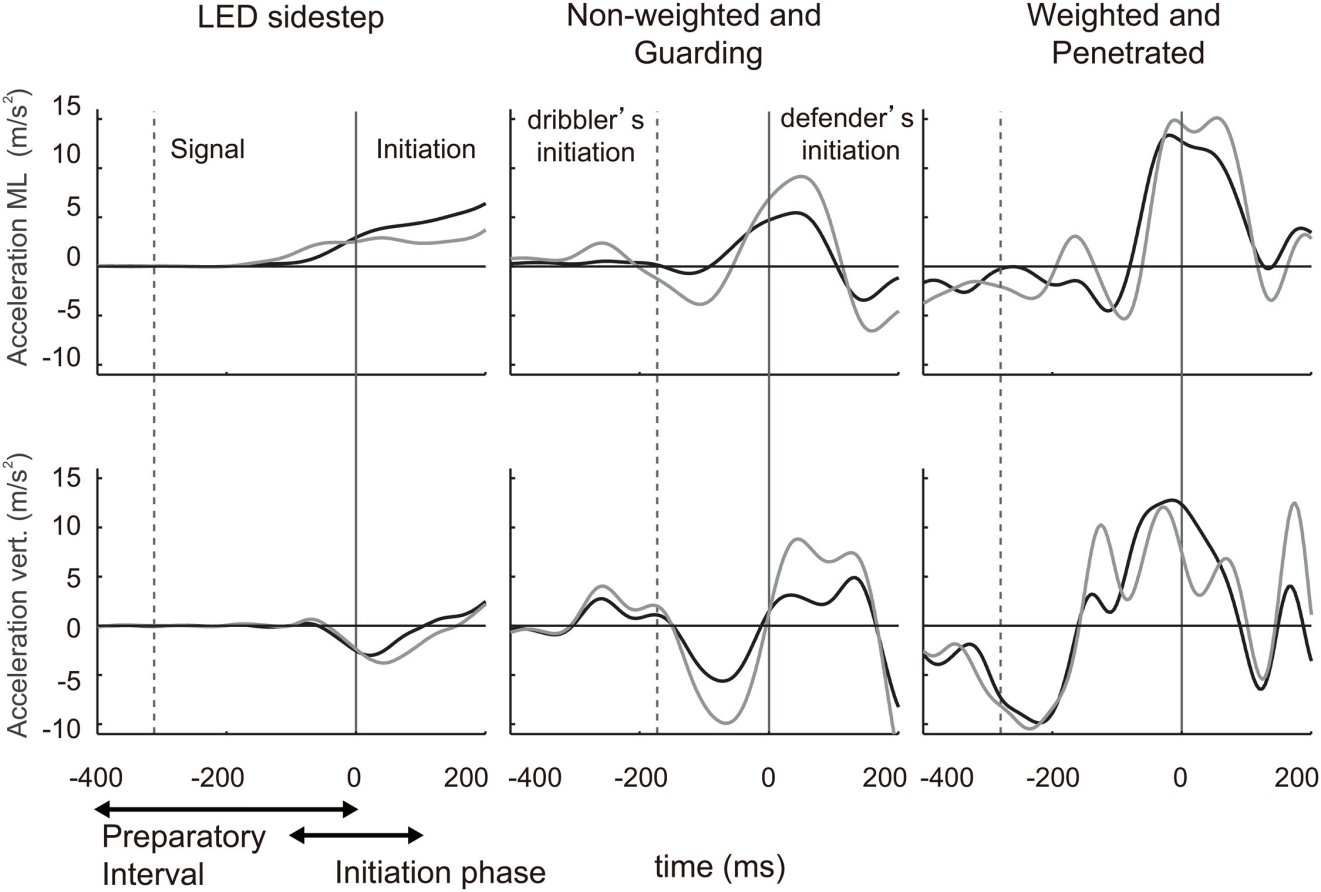
Time series of the trunk and center of mass acceleration. Typical examples of participant’s mediolateral trunk (gray) and center of mass (CoM, black) accelerations (upper panel), and vertical mediolateral trunk and CoM accelerations (lower panel) in a choice-reaction sidestep, a non-weighted (NW) and guarding, and weighted (W) and penetrating trial. The vertical solid and dashed lines are defender’s (0 ms) and choice-reaction signal illumination or dribbler’s initiation times, respectively. Initiation phase is defined as the interval from 100 ms before the defender’s initiation to 100 ms after the initiation.

To investigate the relationship between the preparatory GRF state and the outcome of a 1-on-1 dribble (Fig 1C), we defined the determination phase as the period of 400 ms before the defender’s initiation time. The phase determined the outcome of the 1-on-1 subphase shown as previous studies [13,16]. The defender’s initiation time was defined as the time to the rising of the defender’s mediolateral trunk velocity exceeding 10% of the peak velocity (Fig 1B). The non-weighted state was defined as the state wherein peak vertical GRFs (*Fz*) of both defender’s feet were less than 120% of the BW in the determination phase. The weighted state was defined as the state wherein the peak *Fz* was above 120% BW on either foot in the determination phase. The force threshold was determined based on two points. First, the outcome-GRF relationship was optimal (Table 1) in terms of the most biased tendency (confirmed by chi-squared test) and well-explained (i.e., non-weighted state tended to successfully guard and vice versa). Second, weighted trial above the force threshold (120% BW) before step initiation resulted in the following slow step initiation in our sidestepping [31] and 1-on-1 previous studies [16]. The preparatory interval (400 ms) should be adequate for two reasons [16]. The first is because the defender takes two or three actions (e.g., move left, right and left) if the interval is too long. The second is that an interval that is too short cannot reflect the great variability in the unpredictable dribbler’s movements (including step initiation) as visual cue timing for the defender, compared with the controlled experimental environments. Defender and dribbler performances were defined using trunk velocity according to the rules of basketball [13], which allow the defender to stop the dribbler with the trunk from a head-on position only. Initiation time difference was defined as the defender’s initiation time relative to the dribbler’s initiation time as defined above. The defender’s mediolateral peak trunk velocity was defined as the first peak after the defender’s initiation time. We also calculated the mediolateral peak acceleration of the trunk in the initiation phase as an instantaneous performance in the initiation, which was defined as the interval from 100 ms before the defender’s initiation to 100 ms after the initiation (Fig 2). As an evaluation of mechanical fluctuation, the root mean square (RMS) of the vertical CoM acceleration during the preparatory interval was calculated and normalized by the mediolateral trunk peak velocity because RMS of the CoM acceleration depends on the gait speed [43].

**Table 1:** Number of trials at each vertical GRF threshold.

		Number of trials at each vertical GRF threshold (BW)						
GRF state	Outcome	0.9	1.0	1.1	1.2	1.3	1.4	1.5
Non-weighted	Guard	6	8	12	17	19	19	25
	Penetrate	2	5	5	8	12	15	16
Weighted	Guard	29	27	23	18	16	16	10
	Penetrate	34	31	31	28	24	21	20
χ^2^		0.40	0.97	4.07	5.41	3.18	1.15	5.31
*p*		0.53	0.62	0.13	0.07	0.20	0.56	0.07
Cramér’s *V*		0.07	0.12	0.24	0.28	0.21	0.13	0.27

Number of trials at each vertical ground reaction force (GRF) threshold shown in body weight (BW). There were 36 guarding trials and 37 penetrating trials. GRF state was categorized into non-weighted and weighted state based on each threshold. We selected the threshold of 1.2 BW as optimal in terms of the most biased tendency (confirmed by chi-squared test) and well-explained (i.e., non-weighted state tended to successful guard and vice versa).

#### Kinetic analysis

Kinetic data were calculated only for the trailing leg relative to the direction of the defender’s movement because of the trailing leg’s contribution to the sidestep initiation both in the choice-reaction task and in basketball defense (Fig 1B). To calculate lower extremity kinetics using inverse dynamics with GRF data, we solved motion equations of lower extremity segment model, in which arm kinematics were not used [42]. The local coordinate systems were set to each of the thigh, shank, and foot segments. External/internal rotation of the hip and knee joints (y-axis) corresponds to the longitudinal axis of the thigh and shank segments, respectively. Abduction/adduction of hip joint and valgus/varus of knee joint (x-axis) was defined as the cross product of each y-axis and the vector from lateral to the medial epicondyles of the femur and the lateral to medial malleolus, respectively. Hip and knee extension/flexion (z-axis) were defined as the cross product of the x- and y-axes. Abduction/adduction of ankle joints (y-axis) corresponds to the longitudinal axis of the foot segment. External/internal rotation of ankle joint was defined as the cross product of y-axis and the vector from lateral to medial malleolus, and plantarflexion/dorsiflexion (z-axis) was defined as the cross product of the x- and y-axes. In the present study, we only analyzed hip abduction/adduction, hip and knee extension/flexion, and ankle plantarflexion/dorsiflexion normalized by the participant’s mass (kg). These movements were based on the characteristic changes were observed in the previous sidejump study [37] and the present study. We did not analyze joint angular velocity and power themselves (showed as the typical examples in S1 and S2 Figs, respectively) because there is a relatively small relationship between GRFs and angular velocity compared with joint torque in terms of dynamics (i.e., less comparable with GRFs than joint torque). Actually, joint angular velocity and power also fluctuated in the 1-on-1 dribble, especially in the weighted state but they seem to be not comparable between choice-reaction sidestep and successful defense trials. Instead, we calculated positive and negative joint work by computing the numerical integration of only positive or negative joint powers, respectively [34].

**Fig 3:**
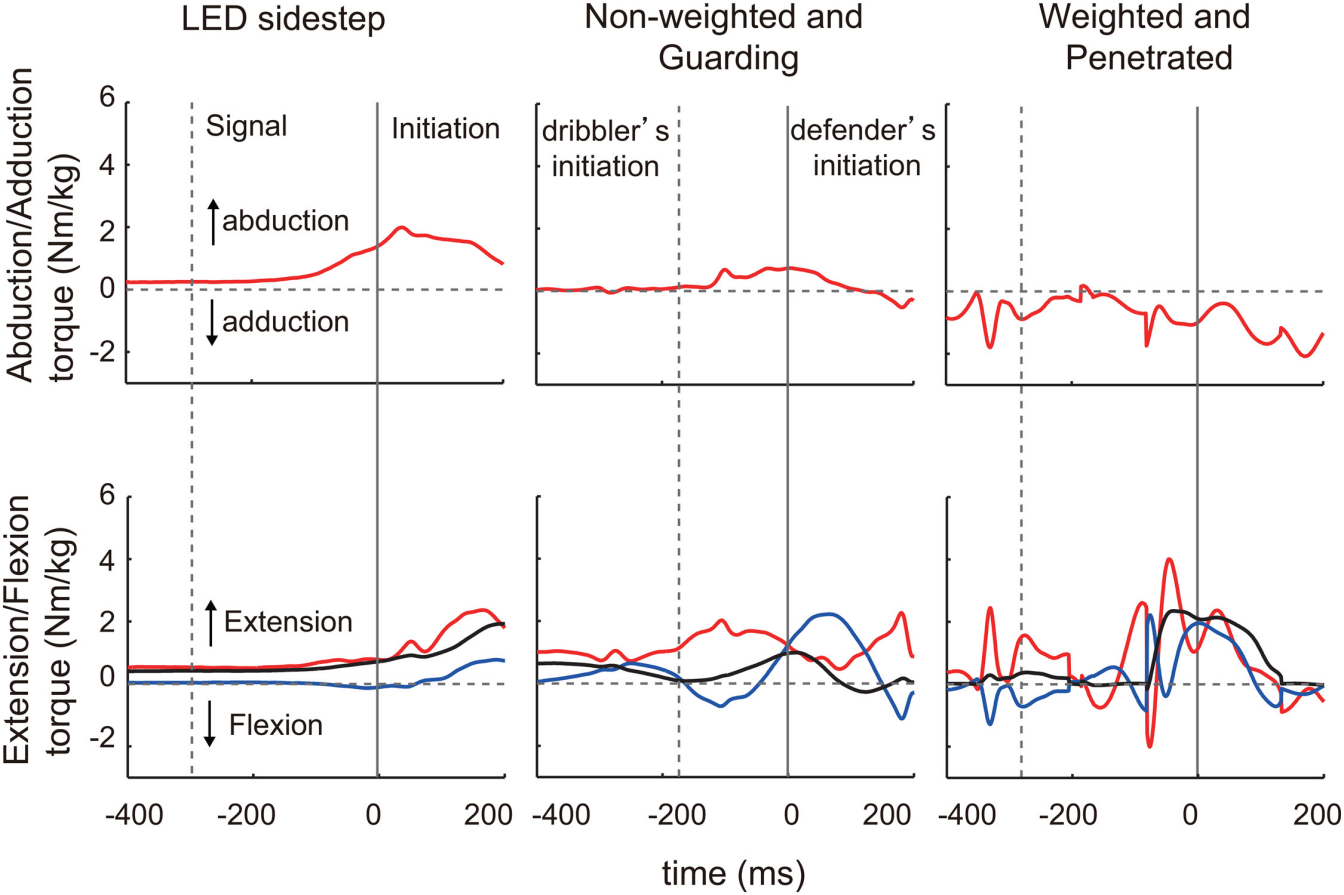
Time series of joint torques. Typical examples of participant’s hip abduction/adduction torque (red, upper panel), and hip (red) and knee (blue) extension/flexion and ankle plantarflexion/dorsiflexion (black) torque (lower panel) in a choice-reaction sidestep, a non-weighted (NW) and guarding, and weighted (W) and penetrating trial. The vertical gray solid lines are defender’s initiation time (0 ms). The vertical dashed lines are choice-reaction signal illumination time in LED sidestep task and dribbler’s initiation time in 1-on-1 dribble task.

For comparisons of kinetic variables between the choice-reaction task and successful defense trials, production time of the joint torque was calculated (Fig 3). This analysis was not performed in the largely fluctuated trials such as in the weighted state. The production time was defined as the time required for each torque to exceed 50% of the local maximal because the torques increased smoothly in this narrow temporal interval. To compare kinetic variables between the outcome and the GRF state in 1-on-1 dribble, the RMS of the time differential of joint torque was calculated to evaluate fluctuation in the determination phase. This method was chosen because the torque oscillated and we were unable to assess the initial rise, especially in the weighted state. Also, we hypothesized that the suppression of the fluctuation in the joint torques could be observed in the guarding trial and non-weighted state based on a previous study in optimal motor control [39].

### Statistical analysis

For comparison of variables between the choice-reaction sidestep and the successful defensive motion, we used unpaired *t*-test if the normality assumption was accepted by Lilliefors Test. If rejected, Mann-Whitney *U*-test was used. To assess the independent and combined effects of the performance and movement characteristics, two-way ANOVAs were used with the two outcomes (penetrating and guarding) and the two GRF states (non-weighted and weighted states), if the hypothesis of homogeneity of variances between groups was accepted with Bartlett’s test. If rejected, the Kruskal-Wallis nonparametric tests were performed to compare these variables. An unpaired *t*-test or Mann-Whitney *U*-test was used to compare the variables within the factor where a significant interaction in ANOVA or a significant effect in Kruskal-Wallis test was found, respectively.

The effect size was estimated using Cohen’s *d* for *t*-test, Cramér’s *V* for Chi-squared test [44] and eta-squared value (*η* ^*2*^) for ANOVA. For the statistical calculations, *p* < .05 was considered significant. All numerical calculations including these statistical analyses were performed using the MATLAB 2011a Statistical Toolbox (The MathWorks, Inc., MA, USA).

## Results

**Fig 4:**
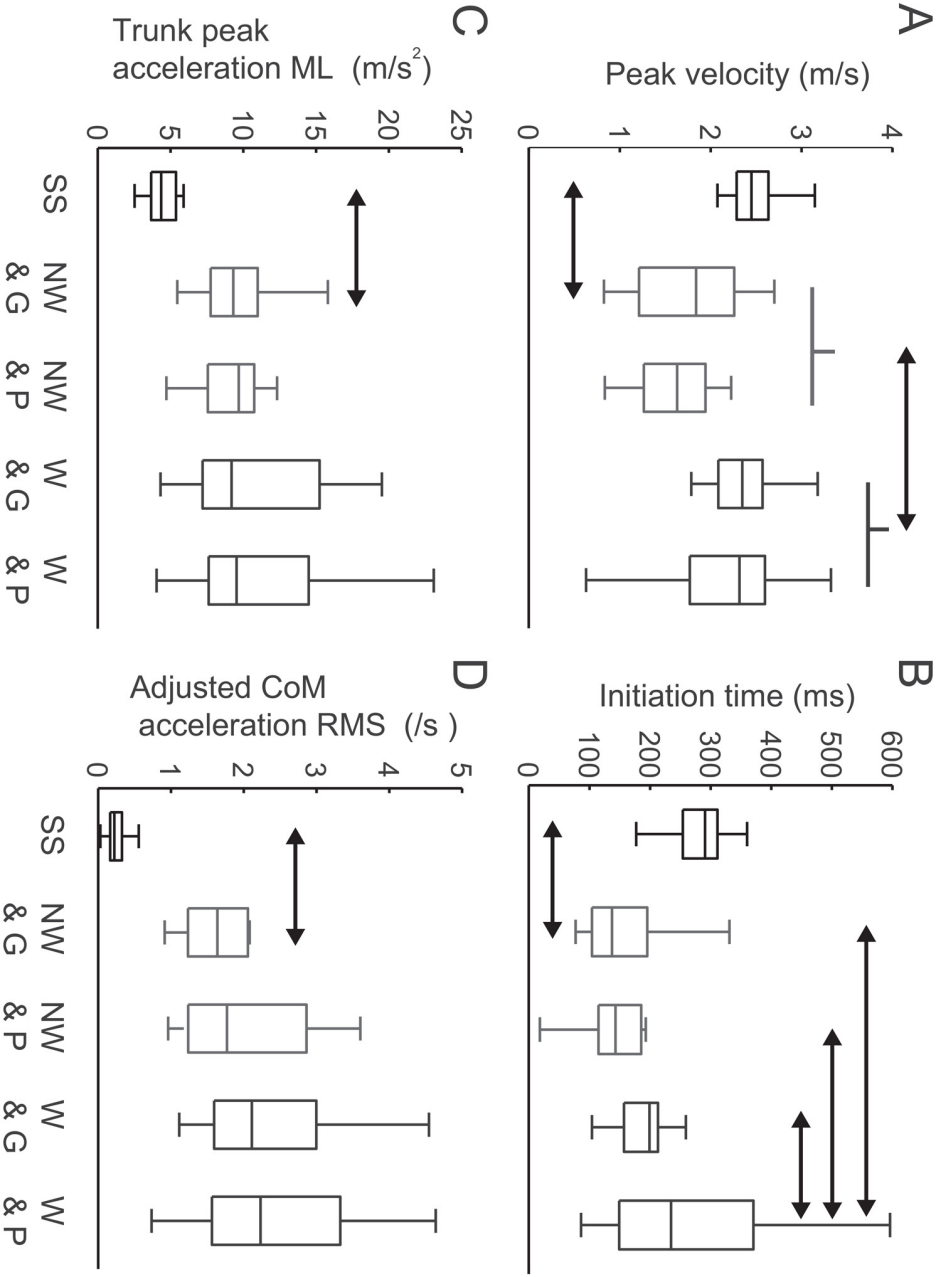
Movement kinematic parameters. Boxplots including median value and the 25th and 75th percentiles in (A) mediolateral trunk peak velocity, (B) initiation time, (C) mediolateral trunk peak acceleration and (D) root mean squares of center of mass acceleration in choice-reaction sidestep normalized by peak mediolateral trunk velocity (SS, black), non-weighted in guarding (NW & G, light gray) and penetrating trials (NW & P, light gray), and weighted in guarding (W & G, dark gray) and penetrating trials (W & P, dark gray). Horizontal arrows represent statistically significant differences between SS and NW & G or between NW and W.

**Fig 5:**
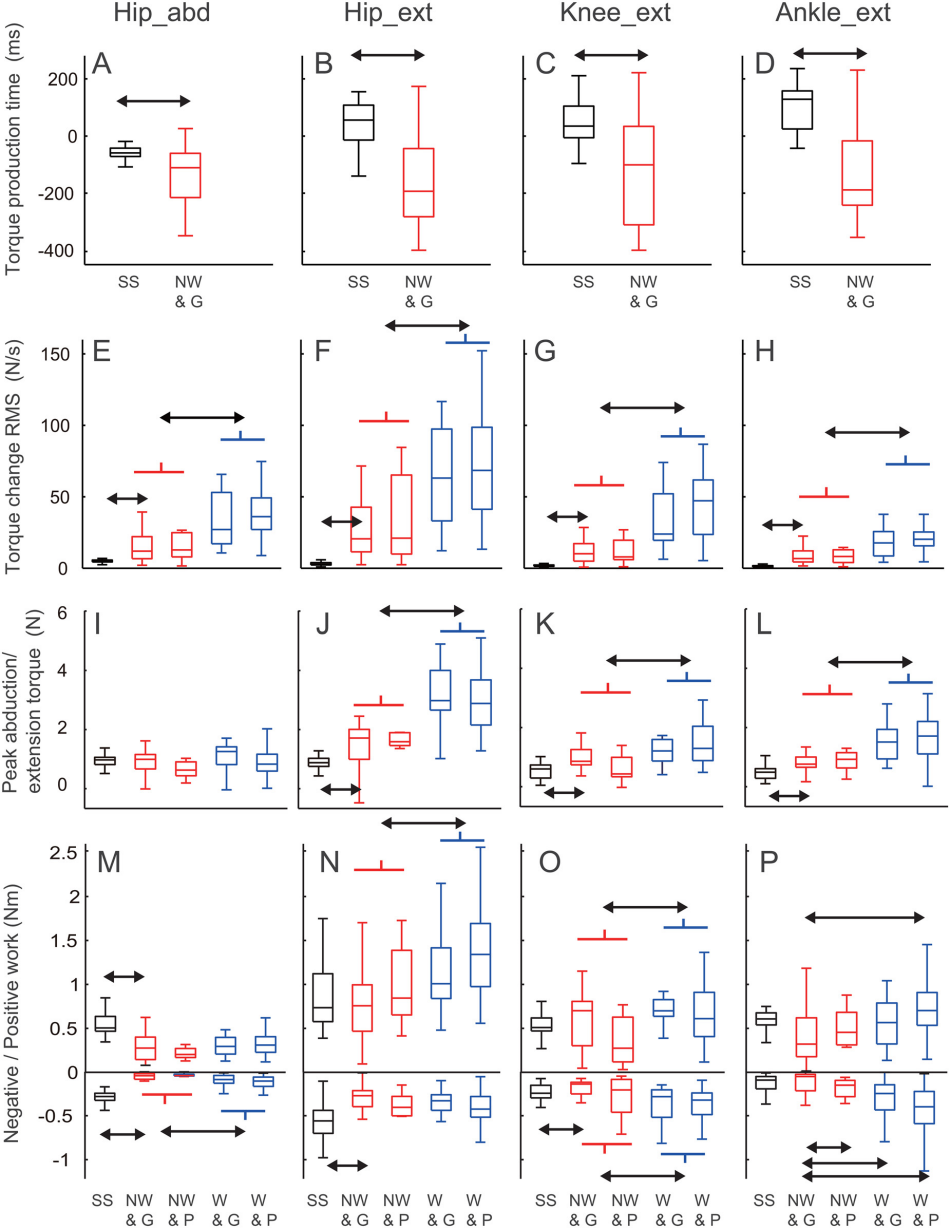
Joint torque parameters. Boxplots including median value and the 25th and 75th percentiles in initiation time of the joint torque (A-D), root mean squares of in time differential of joint torque (E-H), peak joint torques (I-L) and total positive and negative joint works (M-P) in choice-reaction sidestep (SS, black), non-weighted in guarding (NW & G, red) and penetrating trials (NW & P, red), and weighted in guarding (W & G, blue) and penetrating trials (W & P, blue). Horizontal arrows represent statistically significant differences between SS and NW & G or between NW and W. Initiation time of the joint torque was calculated only for comparisons of kinetic variables between sidestepping task and successful defense trial, whereas this analysis were not performed in the largely fluctuated trials such as in the weighted state (Fig 3).

Fig 4 shows the difference in performance between the choice-reaction sidestep and the successful defensive motion in the non-weighted state guarding trials. Although mediolateral peak velocity in the successful defensive motion was slower than that in the choice-reaction sidestep (Fig 4A, *t*_*61*_ = 6.4, *p* = 2.7 × 10^-8^, *d* = 1.9), the initiation time relative to the stimulus in the successful defensive motion was shorter than that in the choice-reaction sidestep (Fig 4B, *t*_*61*_ = 7.5, *p* = 2.8 × 10^-10^, *d* = 2.1). Typical examples of mediolateral velocity are shown in Fig 1B. For kinetics in the choice-reaction sidestep, hip abduction torque was produced first, followed subsequently by extension torques for the hip, knee, and ankle joints (Fig 5A–5D, all *p* < 10^-14^). In the successful defensive motion in the non-weighted state guarded trials, the time required for the production of hip abduction and the three extension torque were significantly shorter (all *p* < 0.01) compared with the choice-reaction sidestep. Furthermore, hip abduction and hip extension torques were produced almost simultaneously (*t*_*13*_ = 0.26, *p* = 0.80, *d* = 0.09). Typical examples of joint torques are shown in Fig 3. The mediolateral trunk acceleration during the initiation phase for defensive motion was larger than that for the choice-reaction sidestep (Fig 4C, *t*_*61*_ = -11, *p* = 4.0 × 10^-16^, *d* = -3.2). For vertical acceleration before initiation, the adjusted RMS in CoM acceleration of the defensive motion was larger than that of choice-reaction sidestepping (Fig 4D, *p* = 3.4 × 10^-19^). Typical examples of accelerations are shown in Fig 2.

Fig 1C shows the results of the categorization into non-weighted and weighted trials for both penetrating and guarding trials. In the determination phase, when the defenders were in the non-weighted state for both feet (S1 Video), they guarded successfully in 68.0% of the trials, whereas in the weighted state (S2 Video) this percentage dropped to 39.1% (*χ* ^*2*^ (1) = 5.4, *p* = 0.067, *V* = 0.28). There was significant difference in initiation time difference among all categories (Fig 4B, *χ* ^*2*^ (67) = 11.0, *p* = 0.012). The defender’s initiation time in the weighted state penetrating trial was longer than the trials in the remaining categories (all *p* < 0.05). For the defender’s mediolateral peak velocity, those in the weighted state were significantly higher than those in non-weighted trials (Fig 4A, *F*_*1*,*67*_ = 10.8, *p* = 1.6 × 10^-3^, *η* ^*2*^ = 0.94). In terms of torque fluctuations, the RMS value of the derivatives of the hip abduction and three extension torques for the hip, knee, ankle joints were significantly different between the outcome and the GRF state (Fig 5E–5H, all *χ* ^*2*^ (67) >17.4, *p* < 10^-3^), however, the values in the non-weighted state were smaller than those in the weighted state (all *p* < 10^-4^), irrespective of the outcome (all *p* > 0.05). Similarly, peak torques in the non-weighted state were smaller than those in the weighted state (Fig 5J–5L,all *p* < 5.0 × 10^-3^) except for the hip abduction (Fig 5I, *p* > 0.05), irrespective of the outcome (all *p* > 0.05). Positive works for the hip and knee extension/flexion and negative works for the hip abduction/adduction and knee extension/flexion were showed similar results (Fig 5M–5O, all *p* < 0.05). Positive work for the ankle extension/flexion in the non-weighted state guarding trial were smaller than that in the weighted state penetrating trial (Fig 5P, *p* = 0.001). Negative works of the ankle extension/flexion in the non-weighted state guarding trial was larger than the trials in the remaining categories (Fig 5P, all *p* < 0.05). There were no significant differences in positive work of the hip abduction/adduction and negative work of hip extension/flexion (Fig 5M and 5N, both *p* > 0.05). The adjusted RMS values of the vertical acceleration of the CoM were not significantly different among all categories (Fig 4D, *p* < 0.05).

## Discussion

### Successful defensive motion in 1-on-1 dribble vs. choice-reaction sidestep

The present study is the first kinetic analysis of a real-time 1-on-1 competitive sport. In the interacting situation, the players can neither predict the environment (opponent’s motion) completely nor at all times control their body to execute the plan (i.e., to defend against dribbler). To compare the motions involved in skill execution where movements must be continually adapted to the changing environment with the motions in relatively controlled environment, we needed to extract the successful motion in terms of both prediction and control. We then extracted the successful defensive motion in 1-on-1 dribble as the non-weighted state guarding trial and compared it with the choice-reaction sidestep task.

In the choice-reaction sidestep task, we showed that hip abduction torque production was followed by the production of extension torques for hip, knee and ankle joints. In a previous voluntarily-started sidejump study, the main role of the hip abduction torque was to maintain the trunk upright before initiation; thereafter, the hip and knee extension and ankle planterflexion torques contributed to produce an initial lateral velocity [34]. The sequence of functions in the choice-reaction sidestep from maintaining an upright posture using hip abduction torque to producing the initial lateral velocity is quite similar to that for the sidejump in the previous study [37]. We then assumed that there could be little difference in the production mechanism of the main torque between the choice-reaction sidestep task and voluntary sidejump.

For the successful defensive motion in non-weighted state guarding trials, in contrast, the times required for the production of hip abduction and three extension torques were significantly shorter than those the choice-reaction sidestep task. Furthermore, the hip abduction and hip extension torques were produced almost simultaneously. The functions of maintaining an upright posture and propulsion in the successful defensive motion were early and executed in parallel, suggesting that the mechanism of executing the functions are completely different from that in the choice-reaction sidestep. Kinematic analysis demonstrated that although mediolateral peak velocity in the successful defensive motion was slower, the initiation time and the mediolateral trunk acceleration during initiation phase was significantly shorter and higher than those for the choice-reaction sidestep, respectively. The successful defensive motion showed preference toward the initiation of motion over the subsequent peak velocity, whereas the fact that the anticipation in defense is frequently executed than that in choice-reaction sidestep [24] should be taken into consideration. The maneuver would emerge only in the more-realistic interactive experimental-setting; in other words, the maneuver in basketball might be different from a maneuver in rugby, in soccer, or in a reaction task to visual stimulus. The adjusted RMS value of vertical CoM acceleration before initiation during defensive motion was larger than that during choice-reaction sidestepping. It suggests that in a successful defensive motion, the short-term fluctuations of the CoM derived from the previous body acceleration or deceleration remained despite the preparatory phase immediately before the objective motion of step initiation (i.e., 400 ms). The present study proposed that an evaluation of the short-term fluctuations can be utilized for the kinetic analysis of interactive tasks in unpredictable environments to understand human motor functions [9, 10] in sports [36, 38] and for the control of multi-joint robots [11, 12].

### Guarding vs. penetrating trials and non-weighted vs. weighted states in 1-on-1 dribble

The second purpose of the present study was to clarify the kinetic and kinematic difference between the penetrating and guarding trials. In regard to the movement performance, the defender’s initiation time in the weighted state penetrating trials was slower than that for the remaining categories, which coincided with our previous 1-on-1 study [16]. Using a preparatory GRF state as a kinetic global variable, defenders in the non-weighted state successfully guarded in 68.0% of the trials in the determination phase, whereas in the weighted state, this percentage dropped to 39.1%. It suggests that the outcome and the preparatory GRF state were related which agreed with the previous sidestepping [31] and 1-on-1 studies [16], thus we focused on the preparatory GRF state in discussion below. The peak velocity in the non-weighted state was significantly slower than that in weighted trials, however, our previous studies suggested that the outcome should be determined before the defender’s time to peak velocity (i.e., peak velocity was not critical factor in 1-on-1 dribble outcome) [16,31]. Combined with our hypothesis that the non-weighted state tended to reduce the delay in initiation and produce the successful defensive outcomes, hence, the non-weighted state would be a desirable preparatory GRF state to prevent a delay in initiation. Competitive movement does not have a clear solution in motor control because of the opponent; thus a global variable such as the preparatory GRF state may be used to explain the kinetic causes of the game outcomes by considering it as dynamical systems [27, 28]. An evaluation using the terms of the preparatory state which refers to the ability to move properly anywhere and anytime, may be useful for understanding highly adaptive motor behavior [6, 26] including coaching sports and controlling multi-joint robots [11, 12] because of the highly redundant control systems which sometimes show complex behavior.

Torque fluctuations as measured by the RMS values of the derivatives of the hip abduction and the hip, knee, and ankle extension torques in the non-weighted state were smaller than those in the weighted state, irrespective of the outcome. In motor control, humans are assume to plan to move by minimizing any change in the joint torques [39]. Our results showed that a greater change in the joint torque in the weighted state will lead to delayed initiation, which would lead to worse performance compared with the non-weighted state. In contrast, the players in the weighted state demonstrated increased fluctuation of joint torques and a time delay in initiation when compared with the non-weighted state. As is the case with the short-term GRF fluctuations from previous actions [16], the short-term torque fluctuations would affect the motor performance. Total joint works in the weighted state for many joints (i.e., positive hip, knee, and ankle extension/flexions and negative hip abduction/adduction and knee and ankle extension/flexions) were larger than those in the non-weighted state. It suggests that the amount of energy in the defender’s motor system at the initiation of the determination phase has a direct impact on the amount of the torque fluctuation. In other words, there was less energy in the system at the initiation of the determination phase in the non-weighted state and thus the less torque fluctuation, which agreed with the peak joint torque in the present study. Therefore, maintaining less joint energy before the step initiation may be important to prevent a delay in initiation and can be considered as the preparatory body state for the step initiation in a competitive sport. For further understanding of the dynamics of the competitive interaction and preparatory body state, a forward simulation study modeling the visco-elasticity component of limb joints [45] implementing anticipatory decision making [24] is required. The present kinetic analysis of adaptive movements in a conflicting environment may contribute to coaching skilled movement, rehabilitation of impairment in motor function, and implementation of movements in multi-joint robotics in a conflicting situation.

Although coaches in sport often focus on a player’s kinematic variables such as foot position and knee and hip angles [46], our previous results emphasize the importance of the preparatory GRF state (i.e., non-weighted state) before the defender’s initiation [16]. In the current study in the non-weighted state before the initiation, the defender’s lower extremity joints suppressed the short-term fluctuations. Furthermore, the defenders must move to the direction where the dribbler moves by maintaining an upright posture and propulsion in parallel during the preparatory phase. The results of the current study suggest that the preparatory body state as explained by short-term joint torque fluctuations before the defensive step would help explain human adaptive behavior in a conflicting environment such as in competitive sports.

## Supporting Information

S1 Data All data used in statistical analysis in Figs 1, 2 and 4 among all categories. (CSV)

S1 Fig Time series of joint powers. Typical examples of participant’s hip power in abduction/adduction direction, hip and knee power in extension/flexion direction and ankle power in plantarflexion/dorsiflexion direction in a choice-reaction sidestep, a non-weighted state guarding, and weighted state penetrating trial. The configuration is the same as Fig 3.
(PDF)

S2 Fig Time series of joint angular velocity. Typical examples of participant’s hip angular velocity in abduction/adduction direction, hip and knee angular velocity in extension/flexion direction and ankle angular velocity in plantarflexion/dorsiflexion direction in a choice-reaction sidestep, a non-weighted state guarding, and weighted state penetrating trial. The configuration is the same as Fig 3.
(PDF)

S1 Video Video clip showing an example of the non-weighted state in a guarding trial. (MP4)

S2 Video Video clip showing an example of the weighted state in a penetrating trial. (MP4)
