# Supplementary figures and images for: Preparatory Body State before Reacting to an Opponent: Short-Term Joint Torque Fluctuation in Real-Time Competitive Sports
Source: PLoS One. 2015 May 29;10(5):e0128571. doi: 10.1371/journal.pone.0128571 (PMC4449124; doi:10.1371/journal.pone.0128571)

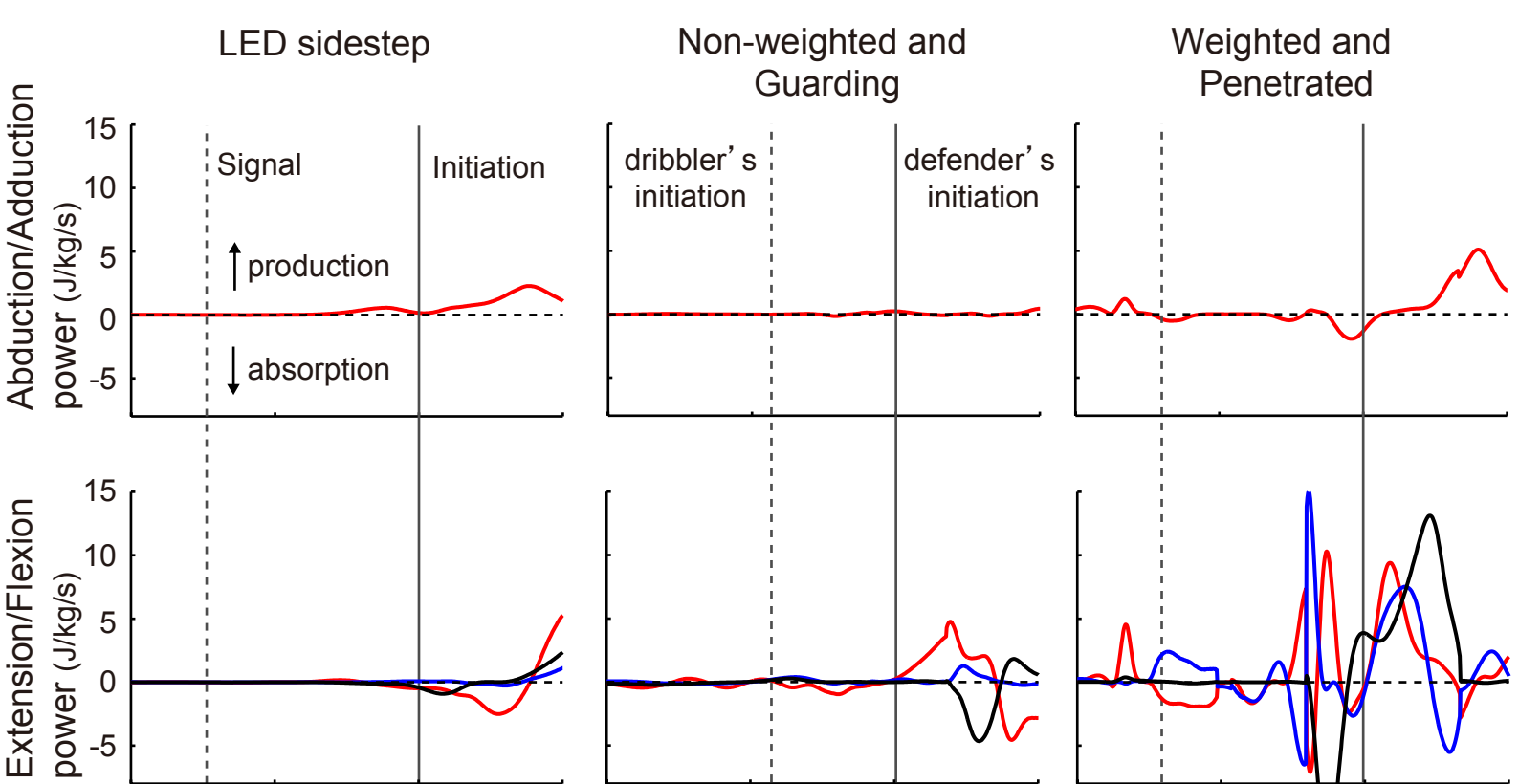

Supplement: S1 Fig — Typical examples of participant’s hip power in abduction/adduction direction, hip and knee power in extension/flexion direction and ankle power in plantarflexion/dorsiflexion direction in a choice-reaction sidestep, a non-weighted state guarding, and weighted state penetrating trial. The configuration is the same as Fig 3. (PDF) [file pone.0128571.s002.pdf]

LED sidestep

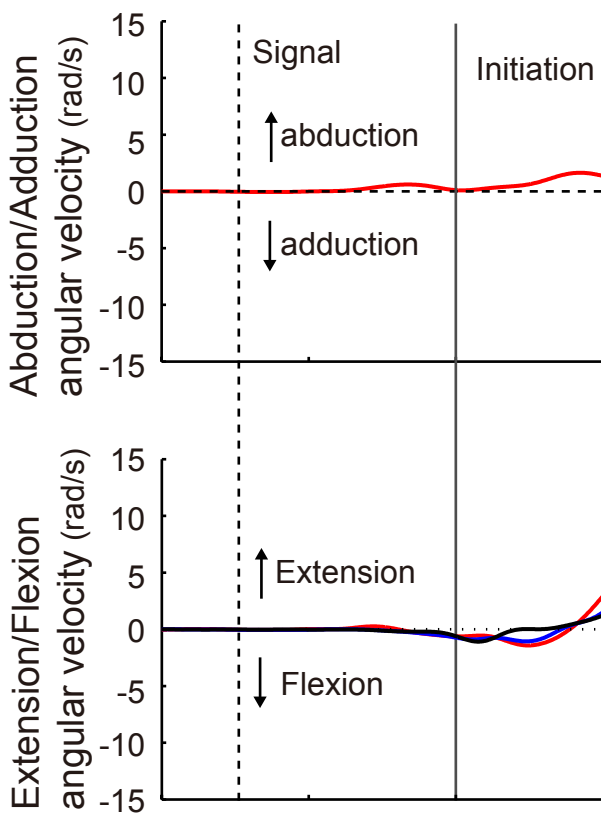

Non-weighted and Guarding

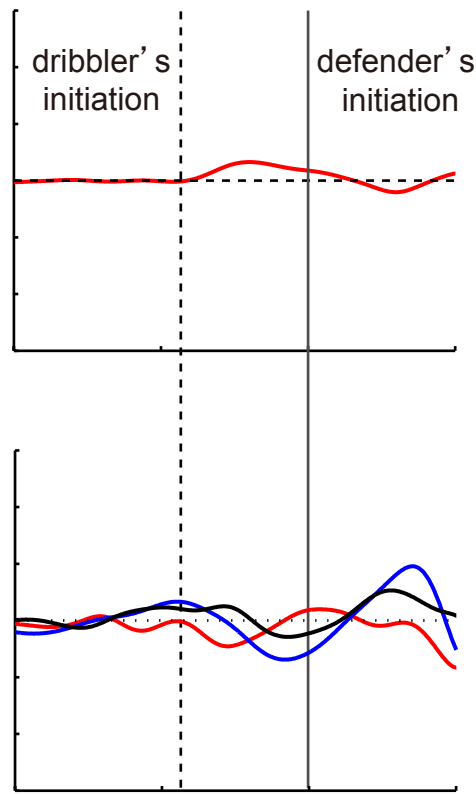

Weighted and Penetrated

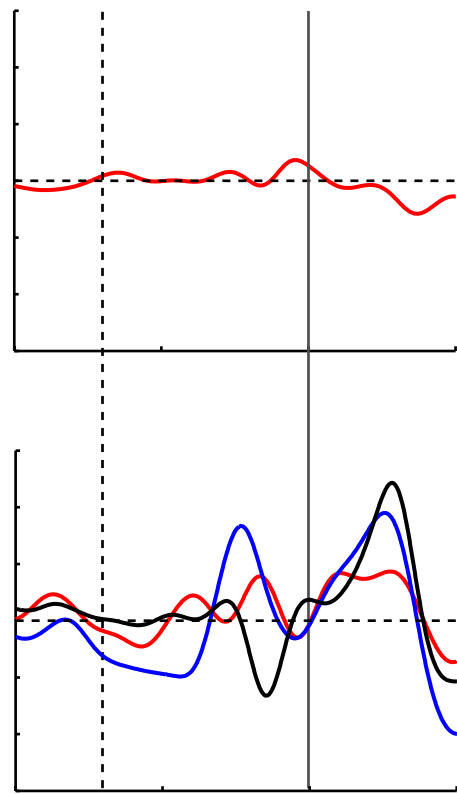

Supplement: S2 Fig — Typical examples of participant’s hip angular velocity in abduction/adduction direction, hip and knee angular velocity in extension/flexion direction and ankle angular velocity in plantarflexion/dorsiflexion direction in a choice-reaction sidestep, a non-weighted state guarding, and weighted state penetrating trial. The configuration is the same as Fig 3. (PDF) [file pone.0128571.s003.pdf]
